# Using life course charts to assess and compare trajectories of amphetamine type stimulant consumption in different user groups: a cross-sectional study

**DOI:** 10.1186/s12954-019-0339-x

**Published:** 2020-01-13

**Authors:** Marcus-Sebastian Martens, Heike Zurhold, Moritz Rosenkranz, Amy O’Donnell, Michelle Addison, Liam Spencer, William McGovern, Roman Gabrhelík, Benjamin Petruželka, Magdalena Rowicka, Nienke Liebregts, Peter Degkwitz, Eileen Kaner, Uwe Verthein

**Affiliations:** 1 Department of Addictology, First Faculty of Medicine, Charles University and General University Hospital in Prague, Prague, Czech Republic; 2 Centre of Interdisciplinary Addiction Research of Hamburg University (ZIS), Department of Psychiatry, University Medical Centre Hamburg-Eppendorf, Hamburg, Germany; 3 Population Health Sciences Institute, Newcastle University, Newcastle upon Tyne, NE2 4AX United Kingdom; 4 Department of Social Sciences, Northumbria University, Newcastle upon Tyne, NE1 8ST United Kingdom; 5 Department of Social Work, Northumbria University, Newcastle upon Tyne, NE7 7XA United Kingdom; 6 Institute of Psychology, Maria Grzegorzewska University, Warsaw, Poland; 7 Bonger Institute of Criminology, University of Amsterdam, Amsterdam, Netherlands

**Keywords:** Life course charts, Amphetamine-type stimulant, Life events, Adults, Life course approach

## Abstract

**Background:**

Amphetamine-type stimulants (ATS) are the second most commonly used illicit drugs in Europe and globally. However, there is limited understanding of what shapes patterns of ATS use over the life course. The ATTUNE project “Understanding Pathways to Stimulant Use: a mixed methods examination of the individual, social and cultural factors shaping illicit stimulant use across Europe” aims to fill this gap. Here we report initial findings from the life course chart exercise conducted as part of qualitative interviews with ATS users and nonusers.

**Methods:**

Two hundred seventy-nine in-depth qualitative interviews were conducted with five ATS user groups (current and former dependent users;current and former frequent users;non-frequent users) and one group of exposed non-ATS users in five European countries (Germany, UK, Poland, Netherlands and Czech Republic). As part of the interviews, we used life course charts to capture key life events and substance use histories. Life events were categorised as either positive, neutral or negative, and associated data were analysed systematically to identify differences between user groups. We applied statistical analysis of variance (ANOVA) and analysis of covariance (ANCOVA) to test for group differences.

**Results:**

Out of 3547 life events documented, 1523 life events were categorised as neutral, 1005 life events as positive and 1019 life events as negative. Current and formerly dependent ATS users showed more negative life events for the entire life course after age adjustment. Although some group differences could be attributed to the individuals’ life course prior to first ATS use, most negative life events were associated with periods of ATS usage. A detailed analysis of the specific life domains reveals that dominantly, the social environment was affected by negative life events.

**Conclusions:**

For non-dependent, frequent and non-frequent ATS users, negative life events from the period of ATS use do not become obvious in our analysed data. Besides preventing a pathway into ATS dependency, the aim of an intervention should be to reduce the harm by for example drug testing which offers also the opportunity for interventions to prevent developing a substance use dependency.

For the group of dependent ATS users, our study suggests holistic, tailored interventions and specialist treatment services are needed, as a single, simple intervention is unlikely to cover all the life domains affected.

## Background

Amphetamine-type stimulants (ATS) refers to a range of drugs including amphetamine, methamphetamine, 3,4-methylendioxy-methylamphetamin (MDMA and ecstasy), fenethylline, ephedrine and prescribed drugs containing methylphenidate (e.g. “Ritalin®”). According to the United Nations Office on Drugs and Crime (UNODC) [1], ATS use is rising rapidly, with seizures of ATS having doubled in the 5 years prior to 2015 (191 tons in 2015). The UNODC estimated that there were 37 million users of amphetamines and 22 million users of ecstasy worldwide in 2015 [1], making ATS the second most commonly used illicit drugs after cannabis [1].

In Europe, as in other parts of the world, a number of different indicators reveal widespread use of amphetamines and ecstasy [2]. The EU drugs agency (European Monitoring Centre for Drugs and Drug Addiction—EMCDDA) in association with the Sewage Analysis CORe group Europe (SCORE), analysed urinary biomarkers in the wastewater of 56 European cities to explore drug-taking habits for amphetamine, methamphetamine and MDMA [3]. The highest level of amphetamine detected in wastewater was found in cities in the north and east of Europe, while methamphetamine levels were highest in cities in the Czech Republic, Slovakia, Eastern Germany and Finland. Belgium, Germany and the Netherlands also reported the highest levels of MDMA use. According to the EMCDDA, among Europeans aged 15–34, the 12-month prevalence of amphetamine use was highest in the Netherlands, at 3.6% [4]. Amphetamine use prevalence was also high in Finland (2.4%), Germany (1.9%) and Czech Republic (1.7%). For the same age group, the 12-month prevalence for MDMA was significantly higher. Again, the highest MDMA 12-month prevalence was found in the Netherlands (7.4%), with high rates of 12-month use also found in Ireland (4.4%), Czech Republic (4.1%), Bulgaria (3.1%) and the UK (2.6%).

A number of previous studies have focussed on increasing our understanding of which factors influence the initiation of amphetamine use [5, 6], and in particular, the transition into methamphetamine [7–11] or ecstasy [12, 13] consumption. Research to date suggests that a range of individual, social and environmental factors affect ATS initiation. These include personality traits, with some evidence to suggest that hedonism, shaped by a curiosity about the ATS effects, sensation seeking and propensity for experimentation, can contribute to initiation of ATS use [5, 11–13]. Furthermore, self-management or coping with mental health problems and trauma have also been identified as a common reason for initiating ATS, especially for methamphetamine users [5–8, 10, 12]. Carbone-Lopez et al’s qualitative study of the experiences of female methamphetamine users living in a Missouri prison found that the majority had experienced critical adverse events in childhood [9]. Family dysfunction (parental mental health problems, domestic violence) and experiences of physical or sexual violence often triggered the transition into the use of methamphetamines for these women. Studies also suggest that prior to ATS initiation, most users have tried other substances, including alcohol, tobacco and marijuana, at a relatively early age (12–14 years) [9, 11, 13]. Social factors also play a key part in influencing ATS initiation, in particular, having friends and/or intimate partners who are already using ATS [6, 7, 9, 10, 12] and/or pressure from close peers [9]. Previous research also highlights functional use of ATS, where consumption is motivated by the desire to increase energy levels to manage the pressures of work, family or life in general [10].

However, whilst several studies have been published exploring factors shaping initiation, there is less available evidence about what affects the subsequent development of ATS consumption over time [14]. A recent review of qualitative literature exploring the individual, social and environmental influences on ATS use concluded that a variety of interrelated factors affected key turning points in drug use trajectories [14]. Both the initiation and continued use of ATS were associated with family, friends and social networks and were linked to individual and social stressors, as well as ongoing health problems and critical life events. Specifically, three major factors have been identified as motivating the continued use of ATS: perceived functionality for stress management, boosting sexual pleasure, clubbing, and reduced insecurity in social situations [9, 15–19]; critical life events such as unemployment, death of a close person, separation from close persons, domestic violence [6, 10, 20]; and withdrawal effects [21–23]. Yet there remains a limited understanding of how ATS use trajectories may vary between different groups of users, the pathways into use and the circumstances which lead to more controlled or more problematic consumption patterns.

The European study “Underst**a**nding Pa**t**hways to S**t**im**u**la**n**t Us**e**: a mixed methods examination of the individual, social and cultural factors shaping illicit stimulant use across Europe” (ATTUNE) seeks to respond to this evidence gap by exploring and comparing the different substance use pathways of five specific ATS user and one non-ATS user groups. Research institutions from Germany, the United Kingdom (UK), Poland, the Netherlands and the Czech Republic formed a consortium to examine interactions between potential influencing factors and the trajectories of the ATS use in all five countries. The research study is the first of its kind as it applies qualitative and quantitative methods to generate an in-depth, contextualised understanding of ATS use over the life course [24].

In this paper, we report on the analysis of life course charts completed as part of semi-structured qualitative interviews with ATS users and non-users across Europe. The aim of this paper was to identify life events associated with the individuals’ substance use trajectory.

## Methods

ATTUNE is a cross-sectional mixed-methods study which seeks to explore the dynamics and trajectories of different ATS use patterns in Europe. In addition to systematic reviews of the existing qualitative and quantitative literature on ATS use, ATTUNE comprises two key components. First are the in-depth semi-structured interviews with ATS users and non-users to explore experiences with ATS over the life course and to identify key turning points in their consumption patterns. Second is the structured questionnaire administered by computer tablet to a larger sample of users and non-users to validate and generalise the qualitative interview findings.

The semi-structured interviews employed two topic guides: one for ATS users and one for non-ATS users who were exposed to ATS (defined as having been present when family or friends took ATS but did not consume themselves and have never taken ATS during their entire life course). The topic guides covered aspects of initiation, continuation and increase and decrease of ATS consumption. As part of these interviews, life course charts were used to help provide a chronological structure for discussions about ATS use over time and to capture more detailed data on participants’ living environment, health conditions, social functioning, life events and broader lifestyle. The life course charts were used here with the very specific intention of providing a more systematic means of recording valuable contextual data. Here we report exclusively on findings from the analysis of the life course data.

Ethical approval for data collection and use was secured in five of the six participating countries, and in the Netherlands, an ethical approval was not required. All participants received an information leaflet about the study, informed consent was then obtained and anonymity and confidentiality protected. The interviews were audio-recorded and transcribed in full. Trained and experienced fieldworkers implemented the interviews that lasted between 60 and 90 min. Participants also received a small incentive after the interview, by way of thanks for their time.

### Life course charts

Life course charts have been used previously in qualitative research as a means of contextualising individual semi-structured interviews [25]. In the substance use field, life course charts are used to assess the intensity of drug use at critical time points in a participants’ history and to observe possible associations between critical life events and changes in the drug use trajectory. One particular study with substance using adolescents found that life course charts enhanced the memory of the respondents to recall their drug career and significant life events in chronological order [26–28]. A review of the use of calendar or timeline instruments demonstrated that these types of instruments contributed to improved data quality as they helped respondents relate specific events and dates to different behaviours and consequences [27].

**Fig. 1:**
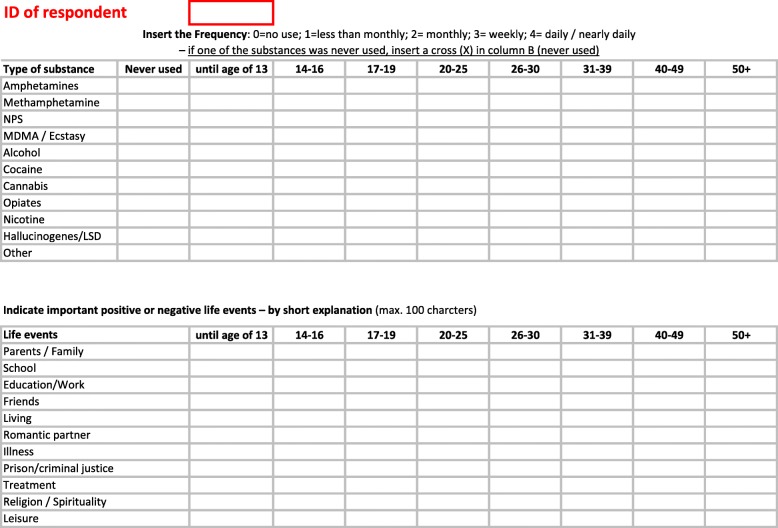
Example of the life course chart used for substances and life events

Timeline instruments have the structure of a chart, with rows referring to the person’s life in years and columns representing different aspects of life (life domains) and substance use, which can vary by study [26, 27]. For this research, a life course chart was developed which reflected the overarching study objectives (see Fig. 1 in Appendix 1). This study used the following defined periods in an individual participants’ life course: until the age of 13, age 14–16, age 17–19, age 20–25, age 26–30, age 31–39, age 40–49 and age 50+. The chart included two separate sections, one referring to substance use and one referring to life events. The section on substances covered the use of different ATS, cannabis, cocaine, opiates and alcohol. The drug use pattern was recorded for each time period by asking users to identify how frequently particular substances were used out of the following five options: no use, use less than monthly, monthly use, weekly use or (almost) daily use. Where the frequency of use varied significantly within one time period, the most frequent use was entered into the life chart. The life event section included 11 different domains, including family history, education, friends, health and illness, involvement with the criminal justice system, substance use treatment and leisure.

The life course chart was implemented as part of the semi-structured, face-to-face interviews with ATS users and non-users. Whilst the life course chart provided interviewers with structured prompts to motivate the interviewer to ask for details of specific live events, the instrument itself was administered iteratively to reflect the fact that certain life events may not have been experienced by some respondents or were not felt to be especially significant. For each of the specific time periods and associated life domains, only one significant life event could be recorded. Where two key life events were reported for the same life domain and time period, the more critical negative life event was noted.

### Sampling criteria and recruitment

To be eligible for inclusion, the first consumption or exposure to ATS needed to have been at least 5 years before the interview took place. Further inclusion criteria were that individuals should be: 18 years or older, have no opioid dependency in their lifetime (with five exceptions for the UK sample), resident in one of the five countries and able to participate in the interview. The inclusion criteria were reviewed with each individual via a standardised screening checklist and only eligible individuals were interviewed.

To ensure variation in ATS pathways and trajectories, we targeted five predefined groups of ATS users and a further group of non-users. Eligible interview participants were allocated to one of the study groups, depending on their consumption pattern, their ATS dependency level and current state of use (Table 1). Dependency was measured with the Severity of Dependence Scale (SDS), which is a five-item questionnaire that provides a score indicating the severity of dependence on amphetamines [29]. An ATS related SDS greater or equal four was approximated as dependency. Former ATS users (defined as not having used ATS in the last 12 months) were asked to fill in the SDS for the phase when their ATS use was the most intense in their life. Current ATS users related the SDS to the last 12 months, and if this result was negative, the SDS was administered again to relate to the most intensive phase.

**Table 1:** Operationalisation of the study groups

Group 1: currently ATS dependent (CDU)	Group 2: formerly ATS dependent users (FDU)	Group 3: currently frequent, non-dependent ATS users (CFU)	Group 4: formerly frequent, non-ATS dependent users (FFU)	Group 5: non-frequent ATS users (currently and formerly) (NFU)	Group 6: exposed non-ATS users (ENU)
SDS positive (last 12 months)	SDS positive (any time except last 12 months)	SDS negative lifetime	SDS negative lifetime	SDS negative lifetime	SDS not applicable
≥ 10 ATS consumption days within past 12 months	≥ 10 ATS consumption days within a year except past 12 months	≥ 10 ATS consumption days within past 12 months	≥ 10 ATS consumption days within a year except past 12 months	< 10 ATS consumption days within any given 12 months	Exposed to ATS without ATS consumption

We aimed to purposively sample five participants per study group in the Czech Republic and 10 participants per group in the other four countries. Participants were recruited into the study using a number of recruitment strategies, including advertising the study by flyer, poster and social media, via contacts within drug and health services, and by interviewees who shared the study link within their networks (snowball sampling). The interviewees were asked how they learnt about the study and more than half of them (51%) were informed by friends or family members. Almost a quarter of participants were recruited by staff of drug treatment services (24%), a smaller number responded to the flyer and posters (16%) and the remaining interviewees heard about the study through social media or from researchers.

In total, 279 eligible participants across five countries participated in the interviews. However, the target sample for each study group could not be realised in all countries (Table 2). While in group 2 (FDU—formerly ATS-dependent users) the recruitment target was exceeded, group 4 (FFU—formerly frequent ATS users) proved difficult to access, especially in Germany and Poland.

**Table 2:** Sample size by country and study group

	Group 1: currently ATS dependent (CDU)	Group 2: formerly ATS dependent users (FDU)	Group 3: currently frequent, non-dependent ATS users (CFU)	Group 4: formerly frequent, non-ATS dependent users (FFU)	Group 5: non-frequent ATS users (currently and formerly) (NFU)	Group 6: exposed non-ATS users (ENU)	Total
Germany	9	17	12	6	9	7	60
United Kingdom	12	14	9	11	11	11	68
Poland	10	10	12	5	15	9	61
The Netherlands	10	10	10	10	10	10	60
Czech Republic	6	5	5	5	4	5	30
Total	47	56	48	37	49	42	279

The interviews were performed by national experienced expert teams. All interviewers were familiarised with the interview materials prior to data collection, all guidance materials were utilised and translated into the national languages where applicable. The Czech partner engaged three well-experienced qualitative researchers, two on post PhD employment; in Germany, the team comprised of three post PhD and two senior researchers; in the Netherlands, experts comprised two persons with experiences in qualitative interviewing, one with MSc and one PhD; in Poland, the team was led by an assistant professor (15 interviews) supported by one experienced university researcher, two experienced external interviewers and two well-trained university co-workers. For the UK, all interviewers were experienced in qualitative interviewing (two post PhD and one MSc) and completed the National Institute for Health Research Good Clinical Practice training.

### Characteristics of the participants

41.2% of the participants were female, and the mean age of all participants was 31 years (Table 3). On average, exposure and use of ATS first occurred when participants were 18 years old. More than a third of participants had, at some point, been in contact with drug treatment services (39.4%), in particular those from group 1 (currently dependent) and group 2 (formerly dependent). SDS screening scores confirmed that participants in group 1 (currently dependent) and group 2 (formerly dependent) were severely dependent, with mean SDS scores of 7.2 and 7.3, respectively [30]. Overall, 33.9% of the sample had ever used ATS (almost) daily, 43.7% of the ATS users consumed (almost) daily or at least weekly amphetamines within the given time periods, but with huge differences among the groups. Daily or weekly amphetamine use was highest in group 1 (currently dependent) at 80.9%, and lowest in group 5 (non-frequent) at 22.4%. In groups 1 (currently dependent) and 2 (formerly dependent), 21.3% and 30.4% took methamphetamines frequently, but no use was reported from group 5 (non-frequent). Daily or weekly MDMA use was evidence in around 30% of the ATS users, but again, group 5 (non-frequent) had the lowest rate of frequent MDMA use.

**Table 3:** Characteristics of the interviewees by study group

	Group 1: currently ATS dependent (CDU)	Group 2: formerly ATS dependent users (FDU)	Group 3: currently frequent, non-dependent ATS users (CFU)	Group 4: formerly frequent, non-ATS dependent users (FFU)	Group 5: non-frequent ATS users (currently and formerly) (NFU)	Group 6: exposed non-ATS users (ENU)	Total
*N*	47	56	48	37	49	42	279
Female	46.8%	44.6%	25.0%	45.9%	40.8%	45.2%	41.2%
Mean age (SD)	32.2 (7.7)	33.6 (8.6)	29.3 (6.8)	32.6 (9.5)	31.0 (7.6)	28.8 (6.8)	31.3 (8.0)
Mean age of onset/exposure ATS use (SD)	16.6 (2.2)	17.6 (4.7)	18.6 (4.0)	17.0 (4.3)	18.8 (3.8)	17.6 (3.4)	17.7 (3.9)
Mean duration of ATS use/exposition in years (SD)	15.5 (8.0)	12.5 (6.8)	10.7 (5.3)	9.9 (6.8)	8.5 (6.5)	11.2 (6.1)	11.5 (6.9)
Ever contact with drug service	68.1%	78.6%	25.0%	29.7%	22.4%	0.0%	39.4%
SDS ATS score (SD)	7.2 (2.8)	7.3 (3.4)	1.9 (1.6)	2.2 (1.8)	0.3 (1.2)	0.0 (0.0)	3.3 (3.8)
Daily ATS use (almost)	74.5%	70.9%	20.8%	22.2%	4.1%	0.0%	33.9%
Daily/weekly amphetamine use	80.9%	71.4%	45.8%	29.7%	22.4%	0.0%	43.7%
Daily/weekly methamphetamine use	21.3%	30.4%	6.3%	10.8%	0.0%	0.0%	12.2%
Daily/weekly MDMA use	44.7%	50.0%	37.5%	35.1%	10.2%	0.0%	30.5%
Ever used cannabis	91.5%	96.4%	91.7%	94.6%	91.8%	78.6%	91.0%
Ever used cocaine	66.0%	62.5%	60.4%	83.8%	51.0%	4.8%	54.8%
Ever daily alcohol use	48.9%	51.8%	27.1%	40.5%	42.9%	28.6%	40.5%

Across all ATS user groups, more than 90% reported lifetime cannabis use, even in group 6 (non-ATS users, at almost 80%). More than half of all respondents reported lifetime cocaine use, with the prevalence highest in group 4 (former frequent) and lowest in group 6 (non-ATS users) at 83.8% versus 4.8%. With regard to alcohol consumption, around 40% of the sample report having ever drunk alcohol on a daily basis, rising to around half of respondents from the two ATS-dependent groups.

### Analysis

A total of 3547 life events were documented in the life course charts for the 279 interviewees, each of which referred to the respective time period when the event occurred. The life events were extracted from the life course charts, with multiple same responses aggregated to a single entry and disconnected from any interviewee information. In order to analyse the life events, experienced researchers based in the respective national research institutions normatively rated each event either as positive, negative or neutral, where neutral means that the life event could not be rated as positive nor negative because according to the normative judgement of the national experts it was neither positive nor negative. As such, the meaning attributed to each event could reflect the cultural importance of certain life events in relation to each national field site, for example, marriage (positive in Poland), living with a partner (neutral in the UK, positive in other countries) and university degree (positive in Germany, neutral in the UK). Table 4 shows examples for the ratings in the defined 11 life domains. None of the interviewees ascribed any positive life event in the categories illness or criminal justice system.

**Table 4:** Examples for ratings of the life events according to the life domain

Life domain	Positive	Negative	Neutral
Parents/family	Caring, supportive parents	Divorce of parents, parental alcohol dependence, domestic violence	Being adopted, in general good family relationships
School	Completed post-compulsory, graduation, certification	Excluded from school, bullying, not completed school	Completed school
Education/work	Started University, regular/proper work	Unemployment, stressful job, dealing drugs	Temporary employment, full-time employment
Friends	Good social network, drug-free friends, having best friends	No friends, social isolation, drug using/dealing friends	Partying or clubbing with friends
Romantic partner	New romantic relationship, marriage, stable partnership	Drug using partner, divorce, separation, domestic violence	Being single, divorce, multiple sex partners
Living	Having own flat, with own family/children	Homeless, kicked-out of home, assisted living	Alone, with parents, with boyfriend, in hostel
Illness		Mental health problems, self-harm, physical injuries	Diagnosed with attention deficit hyperactivity disorder, physical recovery in hospital
Criminal justice system		Imprisonment, conviction, arrests	Drug-related crime, occasional/minor offenses
Treatment	Detoxification, outpatient drug treatment, psychotherapy, psychiatry	In hospital, in psychiatry	Detoxification, psychiatry, rehabilitation
Religion/spirituality	Yoga, meditation	Strong aversion to faith and church	Catholic upbringing, atheist
Leisure	Travelling, playing music, sport	Boredom, social isolation, drug use, always staying at home	Clubbing, festivals, raves

For each person, we calculated the cumulated sum of negative, positive and neutral life events at different points in time for all life domains together, as well as the sum for negative life events for every single life domain. An empty cell in the life course chart for life events was counted for the sum scores as zero. The sums of life events serve in our models as the dependent variable. For the analysis of the life events in the entire life course, we applied an age-adjusted factorial analysis of covariance ANCOVA, with the independent variables study group and country. For the analysis of life events while using ATS, we calculated a duration-adjusted ANCOVA in which for group 6 (non-ATS users) we determined the duration from age at first ATS exposition to the current age. For comparing the six groups (independent variable), we computed univariate analysis of analysis of variance (ANOVA) and ANCOVA in SPSS [31] and chose partial ETA squared as an indicator of the effect size, where 0.01 is considered as small, 0.06 as medium and 0.14 as large effect [32]. A *p* value of < 0.05 was employed to state statistical significance.

These data analysis were largely explorative, but the following hypotheses were guiding some parts of the analysis:

1. Dependent ATS users will show more negative life events in total
2. Dependent ATS users will show more negative life events before the onset of ATS use
3. Dependent ATS users will show more negative life events from the time period using ATS

## Results

Overall, 1523 life events rated as neutral, 1005 life events as positive and 1019 life events as negative.

We did not observe any statistically significant differences between males and females in an age-adjusted ANCOVA for the total positive life events (male, 1.7 (SD = 2.2); female, 1.9 (SD = 2.2)), total neutral life events (male, 2.5 (SD = 3.3); female, 3.0 (SD = 3.3)) and total negative life events (male, 1.6 (SD = 1.8); female, 2.0 (SD = 2.4)). Only the combined sum of positive, neutral and negative life events (male, 5.8 (SD = 4.6); female, 6.9 (SD = 5.3)) showed statistically significant group differences (*F*(1,276) = 3.92, *p* = 0.049).

A two-way age-adjusted ANCOVA was conducted to compare the main effects of the independent variables study group and country and the interaction effects between study group and country on the sum of all four categories of documented life events. The average sum of all documented life events did not differ significantly between the groups, ranging from 13.6 in group 2 (formerly dependent) to 11.4 in group 3 (currently frequent). No significant differences were observed for the neutral life events, which ranged from 6.7 in group 6 to 4.5 in group 3 (Table 5). The average number of positive life events per person was lowest for group 1 (currently dependent), with 2.1 documented positive life events in the entire life course, and highest among group 3 (currently frequent) and group 4 (formerly frequent) at 4.0. However, despite these group differences, the ANCOVA, adjusted for age, failed to show statistical significance.

**Table 5:** In the entire life course: mean number and standard deviation (SD) of life events by groups, two-way ANCOVA (group and country), age-adjusted

	Group 1: currently ATS dependent (CDU)	Group 2: formerly ATS dependent users (FDU)	Group 3: currently frequent, non-dependent ATS users (CFU)	Group 4: formerly frequent, non-ATS dependent users (FFU)	Group 5: non-frequent ATS users (currently and formerly) (NFU)	Group 6: exposed non-ATS users (ENU)	Total	*F*(5,248)	*p*	*η*_*p*_^2^
Positive life events (SD)	2.1 (2.4)	3.9 (3.7)	4.0 (3.7)	4.0 (4.1)	3.7 (5.3)	3.9 (4.4)	3.6 (4.0)	2.19	0.056	0.04
Neutral life events (SD)	5.4 (4.8)	4.5 (4.0)	4.8 (5.1)	6.3 (5.7)	5.6 (5.6)	6.7 (6.0)	5.5 (5.2)	0.97	0.436	0.19
Negative life events (SD)	5.3 (2.9)	5.2 (3.2)	2.7 (2.9)	3.3 (2.5)	2.9 (2.1)	2.1 (2.1)	3.7 (2.9)	9.89	0.000	0.17
All life events (SD)	12.8 (5.9)	13.6 (6.1)	11.4 (7.2)	13.6 (9.7)	12.2 (9.2)	12.7 (7.5)	12.7 (7.6)	0.22	0.955	0.04

Both dependent ATS user groups (CDU and FDU) reported considerably more negative life events than the other four groups. The dependent users (CDU and FDU) appeared on the upper end of cumulated negative life events, whereas the frequent users and the non-frequent users (CFU, FFU and NFU) were in the middle, and the exposed non-ATS users (ENU) showed the lowest number. The age-adjusted ANCOVA showed a statistically significant difference between groups (*F*(5,248) = 9.89, *p* = 0.000). The group effect was large (*η*_*p*_^2^ = 0.17).

The country effects were statistically significant for all four scores of life events: positive life events (*F*(4,248) = 33.57, *p* = 0.000, *η*_*p*_^2^ = 0.35); neutral life events (*F*(4,248) = 28.13, *p* = 0.000, *η*_*p*_^2^ = 0.31); negative life events ((*F*(4,248) = 2.80, *p* = 0.026, *η*_*p*_^2^ = 0.04); all life events ((*F*(4,248) = 9.78, *p* = 0.000, *η*_*p*_^2^ = 0.14).

The interaction effect between study group and country did not become significant for any of the four life event scores: positive life events (*F*(20,248) = 1.58, *p* = 0.058, *η*_*p*_^2^ = 0.113), neutral life events (*F*(20,248) = 1.25, *p* = 0.212, *η*_*p*_^2^ = 0.092), negative life events ((*F*(20,248) = 0.81, *p* = 0.697, *η*_*p*_^2^ = 0.062) and all life events ((*F*(20,248) = 1.333, *p* = 0.159, *η*_*p*_^2^ = 0.097).

In order to investigate negative life events in more depth, the group differences were analysed for each of the life domains in respect of the cumulated sum of negative life events. Highest mean sums for negative life events are reported for the dependent user groups with 1.16 (formerly dependent) and 1.15 (currently dependent) in the domain parents/family. For the domains parents/family, friends, romantic partner and illness, we found that dependent ATS user groups showed higher sums of negative life events over the entire life course in comparison to the four other groups. The ANCOVA, age-adjusted, demonstrates statistically significant group differences with low to medium effect sizes (Table 6). Group differences for other life domains, such as criminal justice system and leisure, fell just short of reaching statistical significance.

**Table 6:** In the entire life course by life domains: mean number and standard deviation of negative life events by groups and life domain, ANCOVA, age-adjusted

Life domain	Group 1: currently ATS dependent (CDU)	Group 2: formerly ATS-dependent users (FDU)	Group 3: currently frequent, non-dependent ATS users (CFU)	Group 4: formerly frequent, non-ATS-dependent users (FFU)	Group 5: non-frequent ATS users (currently and formerly) (NFU)	Group 6: exposed non-ATS users (ENU)	Total	*F*(5, 272)	*p*	*η*_*p*_^2^
Parents/family (SD)	1.15 (1.10)	1.16 (1.11)	0.71 (1.17)	0.81 (0.91)	0.55 (0.84)	0.76 (1.05)	0.87 (1.06)	2.84	0.016	0.05
School	0.15 (0.36)	0.23 (0.43)	0.06 (0.24)	0.11 (0.39)	0.22 (0.55)	0.10 (0.30)	0.15 (0.40)	1.83	0.106	0.03
Education/work	0.23 (0.48)	0.32 (0.47)	0.10 (0.31)	0.19 (0.46)	0.22 (0.47)	0.10 (0.30)	0.20 (0.43)	1.38	0.233	0.03
Friends	0.83 (1.32)	0.55 (0.83)	0.29 (0.54)	0.32 (0.58)	0.39 (0.70)	0.24 (0.58)	0.45 (0.83)	3.12	0.009	0.05
Romantic partner	0.36 (0.79)	0.39 (0.68)	0.19 (0.49)	0.11 (0.31)	0.06 (0.24)	0.07 (0.26)	0.21 (0.54)	4.17	0.001	0.07
Living	0.53 (0.80)	0.48 (0.69)	0.17 (0.43)	0.38 (0.76)	0.45 (0.79)	0.29 (0.92)	0.39 (0.74)	1.20	0.309	0.02
Illness	1.02 (1.15)	1.14 (1.31)	0.73 (1.27)	0.68 (0.88)	0.51 (0.87)	0.45 (0.74)	0.77 (1.10)	3.11	0.010	0.05
Criminal justice system	0.77 (1.11)	0.64 (1.02)	0.27 (0.96)	0.54 (1.14)	0.35 (0.78)	0.12 (0.33)	0.46 (0.95)	2.22	0.053	0.04
Treatment	0.04 (0.20)	0.02 (0.13)	0.02 (0.14)	0.08 (0.28)	0.02 (0.14)	0.00 (0.00)	0.03 (0.17)	1.08	0.373	0.02
Religion/spirituality	0.04 (0.29)	0.00 (0.00)	0.04 (0.20)	0.03 (0.16)	0.04 (0.20)	0.02 (0.15)	0.03 (0.19)	0.39	0.854	0.01
Leisure	0.15 (0.62)	0.23 (0.63)	0.08 (0.28)	0.08 (0.28)	0.04 (0.20)	0.00 (0.00)	0.10 (0.42	1.92	0.090	0.03

The sum of all life events occurring until the first ATS use or exposition did not show group differences. However, there were significant differences between groups for the number of positive, neutral and negative life events (Table 7). Neutral life events were higher in the exposed non-ATS user group (ENU) and fewest in the currently dependent ATS user group (CDU). The ANOVA demonstrated statistically significant differences between both groups (*F*(5,273) = 3.29, *p* = 0.007). The positive life events revealed a similar trend for the groups (*F*(5,273) = 2.27, *p* = 0.048). The combined sum of negative life events occurring before first use or exposition to ATS were highest for the two dependent groups, lower for the non-dependent user groups, and lowest of all for the non-users (*F*(5,273) = 2.37, *p* = 0.039). However, the group size effects on the observed differences in positive, neutral and negative life events are small.

**Table 7:** Until the first ATS use/exposure: mean number and standard deviation (SD) of life events, ANOVA

	Group 1: currently ATS dependent (CDU)	Group 2: formerly ATS dependent users (FDU)	Group 3: currently frequent, non-dependent ATS users (CFU)	Group 4: formerly frequent, non-ATS-dependent users (FFU)	Group 5: non-frequent ATS users (currently and formerly) (NFU)	Group 6: exposed non-ATS users (ENU)	Total	*F*(5, 273)	*p*	*η*_*p*_^2^
Positive life events (SD)	0.40 (0.88)	1.07 (1.74)	1.38 (1.61)	1.19 (2.42)	1.67 (3.15)	1.60 (2.12)	1.21 (2.11)	2.27	0.048	0.040
Neutral life events (SD)	1.55 (1.53)	1.38 (1.67)	2.13 (2.77)	2.62 (2.88)	2.94 (3.72)	3.17 (3.75)	2.24 (2.87)	3.29	0.007	0.057
Negative life events (SD)	1.96 (1.88)	2.05 (1.73)	1.42 (2.02)	1.65 (1.72)	1.33 (1.39)	1.07 (1.39)	1.60 (1.73)	2.37	0.039	0.042
All life events (SD)	3.91 (3.05)	4.50 (3.26)	4.92 (4.12)	5.46 (5.65)	5.94 (6.27)	5.83 (4.96)	5.05 (4.65)	1.39	0.228	0.025

The duration of ATS use for the five user groups and the exposure to ATS for the non-ATS user group (ENU) showed group differences (Table 3). Therefore, we applied an ANCOVA adjusted by the duration of ATS use to test for group differences with regard to life events in the time between the onset and desistance of ATS use (Table 8)

**Table 8:** Continued ATS use: mean number and standard deviation (SD) of life events by groups, ANCOVA, duration-adjusted

	Group 1: currently ATS dependent (CDU)	Group 2: formerly ATS-dependent users (FDU)	Group 3: currently frequent, non-dependent-ATS users (CFU)	Group 4: formerly frequent, non-ATS-dependent users (FFU)	Group 5: non-frequent ATS users (currently and formerly) (NFU)	Group 6: exposed non-ATS users (ENU)	Total	*F*(5, 272)	*p*	*η*_*p*_^2^
Positive life events (SD)	1.7 (2.0)	2.0 (2.2)	1.9 (1.9)	1.5 (1.6)	1.5 (2.5)	2.2 (2.8)	1.8 (2.2)	0.73	0.605	0.01
Neutral life events (SD)	3.8 (4.3)	2.5 (3.1)	2.2 (3.2)	2.5 (2.7)	1.8 (2.4)	3.5 (3.5)	2.7 (3.3)	2.98	0.012	0.05
Negative life events (SD)	3.3 (2.5)	2.6 (2.7)	0.9 (1.0)	1.4 (1.6)	0.9 (1.2)	1.1 (1.3)	1.7 (2.1)	13.16	0.000	0.20
All life events (SD)	8.8 (5.1)	7.1 (5.4)	5.0 (3.9)	5.4 (4.2)	4.2 (4.6)	6.8 (4.7)	6.2 (4.9)	5.75	0.000	0.10

The reported sum of all life events while using ATS differs significantly between groups, ranging from 4.2 (SD = 4.6) in group 5 (non-frequent) to 8.8 (SD = 5.1) in group 1 (currently dependent). The number of neutral life events also differed significantly. The positive life events showed no group effects. The negative life events while using ATS were comparably low in the four non-dependent groups, but up to 3.7 times higher for the two dependent groups (CDU and FDU). The lowest number of negative life events were found among group 5 (NFU) with 0.9 while using ATS, and the highest in group 1 (CDU) with 3.3. The duration adjusted ANCOVA showed statistically significant differences between the groups (*F*(5,272) = 13.16, *p* = 0.000). The partial ETA squared demonstrated a large size effect (*η*_*p*_^2^ = 0.20).

## Discussion

Findings from our analysis of drug use and life event data from 279 ATS user and non-users across Europe demonstrate clear differences amongst our six pre-defined user groups, both in terms of the types of ATS used, and in relation to patterns of consumption (Table 2). Intensive, daily ATS use was evident in groups 1 and 2 (dependent—CDU and FDU) and indicative of the overall severity of their drug “career”. In contrast, this consumption pattern was considerably less common in groups 3 and 4 (frequent—CFU and FFU). The type of ATS consumed was also associated with severe (or dependent) use trajectories. Specifically, we found that methamphetamine use was markedly more prevalent amongst current or former dependent user (group 1 and 2 compared to groups 3, 4 and 5), involving periods of daily or at least weekly consumption. We found similar patterns of intensive use amongst groups 1 and 2 (dependent—CDU and FDU) for other non-amphetamine substances, which again contrasted with that reported by our other user groups.

Whilst few differences were seen in the total number of life events reported by different ATS user and non-user groups, we found significant associations between the number and type of negative life events and ATS consumption trajectories. Exposed non-users and non-dependent ATS users, regardless of the frequency of use, reported lower rates of adverse life events than dependent users.

Dependent users, both current and former, reported higher rates of negative life events before their first use of ATS, and fewer positive or neutral experiences. Although small, these differences were nevertheless statistically significant and may suggest that dependent users are more likely to have experienced difficulties during childhood and adolescence.

Negative life events after the initiation of ATS use can be partially interpreted as consequences of the substance use patterns and our results point out that these numerous consequences are exclusively limited to the dependent or formerly dependent users of group 1 and group 2. We do not find any significant differences between the other four groups.

At the same time, the negative life events experienced by dependent ATS users appeared to derive from a variety of life domains, making it challenging to identify clear and causal pathways. Whilst existing evidence implies that experiencing negative life events results in sustained ATS consumption [6, 10, 20], our study suggests holistic, tailored interventions and specialist treatment services are needed for this group, as a single, simple intervention is unlikely to cover all the life domains affected. A standardised short screening tool for life domains affected by negative life events for dependent ATS user in contact with drug services could serve as a guiding action for further support.

For frequent and non-frequent ATS users negative life events from the period of ATS use do not become obvious in our analysed data. Besides preventing a pathway into ATS dependency, the aim of an intervention should be to reduce the harm from the illicit drug itself. Evidence suggests this can be achieved by offering quality and quantity control through drug testing which is well accepted by the users and offers the opportunity for interventions to prevent developing a substance use dependency [33].

Overall, our findings show that formerly or current ATS dependent users are more likely to have experienced higher numbers of negative life events compared to other user groups. However, at present, there is no standardised instrument available to support accurate measurement of negative life events. The development and implementation of such an instrument would be helpful in counselling and treatment settings to provide adequate responses to clients' needs.

## Strengths and limitations

The whole spectrum of ATS careers including ongoing and former use, and ranging from dependent use to non-dependent use; from frequent to non-frequent use, as well as non-use, and experienceor no experience with drug treatment, are represented in the sample and the six groups.

In order to reveal more accurately the context of “change” for ATS trajectories, we established the inclusion criterion “ATS abstinence in the last 12 months” for group 2 (FDU—formerly ATS-dependent users) and group 4 (FFU—formerly frequent, non-ATS-dependent users).

The use of a calendar technique such as timelines [34] for the combination of life events and time points is novel and understudied in the field of substance use in general [26, 35, 36] has yet not been used for examining stimulant use and might tackle partially the individual recall bias of life events.

ATS covers a variety of different substances and the often-observed use of more than one type of ATS in life by the respondents has not been explored in detail in this study as well as the effect of a single ATS. We also have significantly more methamphetamine users in the dependent user groups (CDU and FDU); therefore, we cannot rule out that some results are influenced by the specific experiences of this user group. The same can be said in relation to other poly-substance use and non-amphetamine substances consumed by the respondents. Although further results from the ATTUNE project should disclose such interactions.

The age periods in the life course charts utilised were relatively large. We also only recorded one life event per domain therefore multiple occurrences of life events could have occurred and could have gone unrecorded. This could have resulted in both the underreporting of the sum of life events per domain but also in total. Further, our focus on negative life events might have concealed consideration of positive life events that serve as protective factors.

Whilst our sample was relatively substantial (*n* = 279), we cannot fully adjust for differences such as country, ATS consumed or other, may be important, participant characteristics due to small numbers per specific group/variable.

The life chart data analysed was collected during semi-structured qualitative interviews and quasi-isolated from the rich in-depth interview data. The sample size, the systematic and the number of recorded life events in the life charts gave us the opportunity to employ standardised methods to test for statistical inferences, although such a method is rather untypical for in-depth qualitative interview data.

## Conclusions

By purposeful sampling of five ATS user groups and one ATS-exposed non-user group, we were able to study the association between ATS pathways and life events in the entire life course. The data was systematically collected using life course charts to capture key life events and substance use histories during in-depth qualitative interviews. The applied method is novel for the examination of ATS trajectories.

Dependent ATS users experienced more negative life events for the entire life course after age adjustment. Whilst, some of the group differences found could be attributed to the life course prior to ATS use, most negative life events resulted from periods of ATS usage. A detailed analysis of the specific life domains reveals that the social environment was most likely to be that affected by the negative life events. No difference between the groups of non-dependent, frequent and non-frequent ATS users and exposed non-ATS users were found.

For non-dependent, frequent and non-frequent ATS users, negative life events from the period of ATS use do not become obvious in our analysed data. Besides preventing a pathway into ATS dependency, the aim of an intervention should be to reduce the harm by for example drug testing which offers also the opportunity for interventions to prevent developing a substance use dependency.

For the group of dependent ATS users, our study suggests holistic, tailored interventions and specialist treatment services are needed, as a single, simple intervention is unlikely to cover all the life domains affected.

## Data Availability

The dataset generated and analysed during the current study is in the ownership of the ATTUNE research group and is available from the corresponding author on reasonable request.

## Abbreviations

ANCOVA: Analysis of covariance
ANOVA: Analysis of variance
ATS: Amphetamine-type stimulant
ATTUNE: “Underst**a**nding Pa**t**hways to S**t**im**u**la**n**t Us**e**: a mixed methods examination of the individual, social and cultural factors shaping illicit stimulant use across Europe”
CDU: Currently ATS dependent (group 1)
CFU: Currently frequent, non-dependent ATS users (group 3)
EMCDDA: EU drugs agency: European Monitoring Centre for Drugs and Drug Addiction
ENU: Exposed non-ATS users (group 6)
FDU: Formerly ATS dependent users (group 2)
FFU: Formerly frequent, non-ATS dependent users (group 4)
MDMA: 3,4-Methylendioxy-methylamphetamin (also ecstasy)
NFU: Non-frequent ATS users, currently and formerly (group 5)
SCORE: Sewage Analysis CORe group Europe
SDS: Severity of Dependence Scale
UK: United Kingdom of Great Britain and Northern Ireland
UNODC: United Nations Office on Drugs and Crime
